# Bronchial artery to pulmonary artery fistula initially misdiagnosed as pulmonary embolism: A case report

**DOI:** 10.1016/j.ijscr.2024.109246

**Published:** 2024-01-11

**Authors:** Shivam Khatri, Steven Epstein, Rooshi Parikh, Brian Bobby Chiong

**Affiliations:** a CUNY School of Medicine, New York, NY 10031, United States of America; b Department of Radiology, St. Barnabas Hospital, Bronx, NY 10457, United States of America

**Keywords:** Case report, Bronchopulmonary arterial fistula, Embolization, Pulmonary embolism

## Abstract

**Introduction:**

Bronchopulmonary arterial fistulas have been reported following lung transplant, and in association with COPD, trauma, radiation therapy, and infection. They may also arise congenitally. Embolization is the most frequent treatment.

**Case presentation:**

We present a case of a 58-year-old male with a prior history of pulmonary tuberculosis who initially presented with minimal hemoptysis for several months. Right upper lobe bronchial artery to pulmonary artery fistulas were discovered by angiography. These were excluded by particle and microcoil embolizations.

**Clinical discussion:**

Relatively unopacified blood from bronchial artery enters right pulmonary artery and causes ill-defined hypodensities mixing with opacified blood, especially compared to uniformly, brightly enhancing left pulmonary artery. As a result, interpreters will frequently incorrectly conclude that right pulmonary artery embolism exists rather than a bronchopulmonary arterial fistula.

**Conclusion:**

In most cases, bronchopulmonary arterial fistulas are treated by bronchial artery embolization; however, direct puncture or stent grafting are alternate considerations depending on the patient's anatomy. In all instances, a multidisciplinary approach is a must.

## Highlights

• Bronchopulmonary artery fistula (BPAF) occurs between the bronchial artery and pulmonary artery.
• Bronchial arteries arise from the aorta, so they are usually underopacified with CTA protocol to rule out PE.
• Embolization is most commonly utilized to treat BPAF.
• Other interventions include lobectomy, stent graft placement and percutaneous direct puncture for the treatment of BPAF.

## 1 Introduction

Massive hemoptysis is most commonly due to bronchiectasis [1]. Bacterial infection and pulmonary tuberculosis are less frequently implicated. Mortality rates range from 6.5 % to 38 % [1]. In approximately 90 % of cases, bleeding is due to erosions of the bronchial artery branches [1]. The next most common bleeding source is from pulmonary arteries followed by the aorta [1]. Pulmonary arteriovenous malformations (PAVM) rarely precipitate large-volume hemoptysis. 4 % of PAVMs involve a systemic or bronchial rather than pulmonary arterial supply [[2], [3], [4]].

Bronchopulmonary arterial fistulas (BPAF) are reported in 2.6–13 % of cases of hemoptysis [5,6]. Bronchial artery communicates with pulmonary artery, pulmonary vein, azygos vein, their branches or tributaries [5,6]. These fistulas are commonly associated with lung transplant conditions such as severe COPD, malignancy, pulmonary fibrosis, trauma, radiation therapy, and perforations complicating pulmonary arterial catheterization (such as Swan-Ganz catheter insertion) [[5], [6], [7]]. Tuberculosis is the leading infectious cause of fistula formation [7]. Congenital etiology and BPAF following lymphoma treatment are rare [5,8].

Making the diagnosis and mapping arterial communications can be quite challenging [7]. Digital subtraction angiography (DSA) is routinely performed [7]. CT angiography (CTA) is also commonly utilized. CTA offers additional information including features of vascular architecture [7].

Bronchopulmonary artery fistula involves flow from bronchial to pulmonary artery segments. Unopacified blood potentially mixes with opacified blood during CTA. This can result in prominent unilateral pulmonary artery filling defects [9]. BPAF can therefore be misdiagnosed as pulmonary embolism. We present a unique case of a patient with bronchial artery to pulmonary artery fistula.

## 2 Case presentation

A 58 year-old male presented to our emergency department (ED) with massive hemoptysis and epistaxis. His history included ethanol use, treated tuberculosis and atrial fibrillation. He was intubated and underwent emergent right bronchial artery Embosphere particle (Merit Medical Systems, South Jordan, UT, USA) embolization (ten vials). Bleeding was halted. He recovered and was discharged home.

Ten months later, he returned to ED because of continual episodes (previous two days) of non-massive bright red hemoptysis. He denied recent trauma, fever or chills. He did describe mild midsternal chest discomfort and exertional dyspnea. He had visited Panama three months earlier. His initial blood pressure was 149/109. Heart rate and lab values were normal. Electrocardiogram demonstrated no change from his previous hospital visit. No clots were detected by transthoracic echocardiography. Cardiology recommended not to start anticoagulants, and to continue his remaining medication regimen.

### 2.1 Investigations

**Fig. 1:**
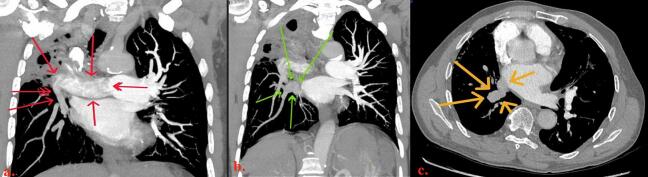
58-year-old male who experienced minimal hemoptysis for which investigations revealed the presence of a bronchopulmonary artery fistula successfully treated with coil embolization. a) Hypoattenuated right pulmonary artery (red arrows). Coronal slices (a and b) depict chronically diseased right upper lobe. b and c) Right pulmonary vein is also under opacified (green and yellow arrows) relative to brightly enhancing vascular structures on the left side.

Chest CTA revealed ill-defined hypodense areas predominantly in the right upper lobe pulmonary artery while main and left pulmonary arterial segments enhanced uniformly. Relative hypodensities were also seen in the right lower lobe and upper lobe arterial branches (Fig. 1).

Pulmonary embolism was therefore initially suspected. No DVT was discovered on follow up ultrasound examination.

**Fig. 2:**
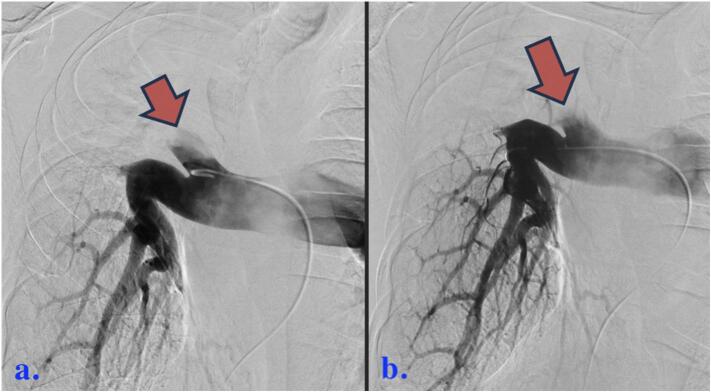
58-year-old male who experienced minimal hemoptysis for which investigations revealed the presence of a bronchopulmonary artery fistula successfully treated with coil embolization. a and b) Right femoral vein puncture. Right upper lobe pulmonary artery initially selected, then interlobar artery. Defect in right pulmonary artery proximal to these branches due to rapid, reversed inflow (red arrows) from unopacified upper lobe branch.

Arteriography using 50 ml contrast at 3 mL/s was undertaken several hours following patient presentation to the ED. Omni Flush catheter (AngioDynamics, Latham, NY, USA) was advanced to the right pulmonary artery through a 5F sheath following right femoral vein puncture. Arteriography demonstrated filling defects due to reversal of flow (Fig. 2).

No clot was detected and no local resistances were encountered during routine coaxial maneuvers. The catheter was advanced to the right upper lobe branch. Local and rapid retrograde flow was again identified both during fluoroscopic contrast injections and in formal arteriography.

### 2.2 Treatment

**Fig. 3:**
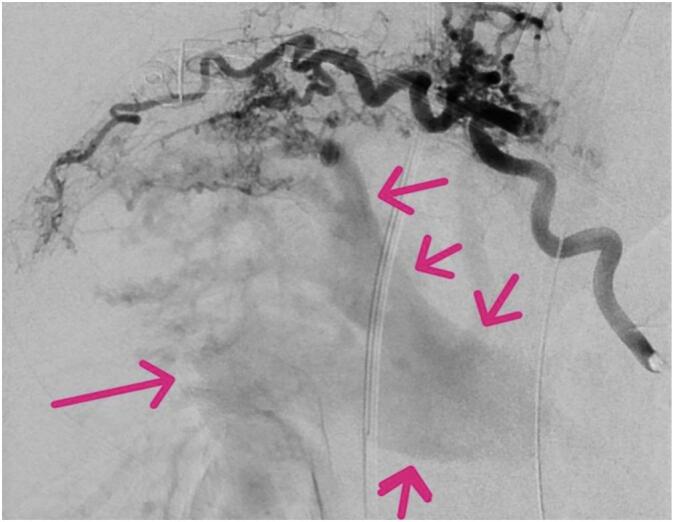
58-year-old male who experienced minimal hemoptysis for which investigations revealed the presence of a bronchopulmonary artery fistula successfully treated with coil embolization. Bronchial arteriogram ten months earlier. Pink arrows demonstrate right pulmonary artery opacifying via communicators arising from bronchial artery branches. Initially treated only with particle embolization which successfully halted massive hemoptysis and from which no clinical worsening was encountered.

**Fig. 4:**
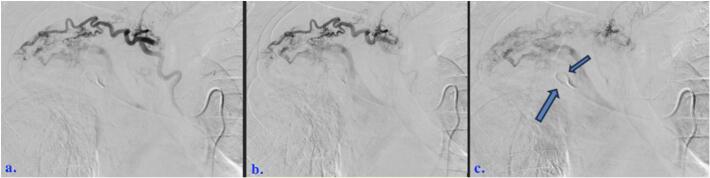
58-year-old male who experienced minimal hemoptysis for which investigations revealed the presence of a bronchopulmonary artery fistula successfully treated with coil embolization. (a through c) One of two right bronchial arteries selected and arteriography performed. Numerous pathologic communicators persist to the right pulmonary artery. Right pulmonary artery flush catheter also present (arrows, c).

The interventional radiologist thereby recognized bronchopulmonary artery fistula while ruling out pulmonary embolism. The right bronchial artery origin was selected with a Mik catheter (Cook Medical, Bloomington, IN, USA) through a 5F vascular sheath following right femoral artery puncture. Angiography demonstrated several prominent first, second and third order arterial branches bridging and shunting to the right upper lobe pulmonary artery (Fig. 3, Fig. 4), and innumerable higher order tortuous communicators.

**Fig. 5:**
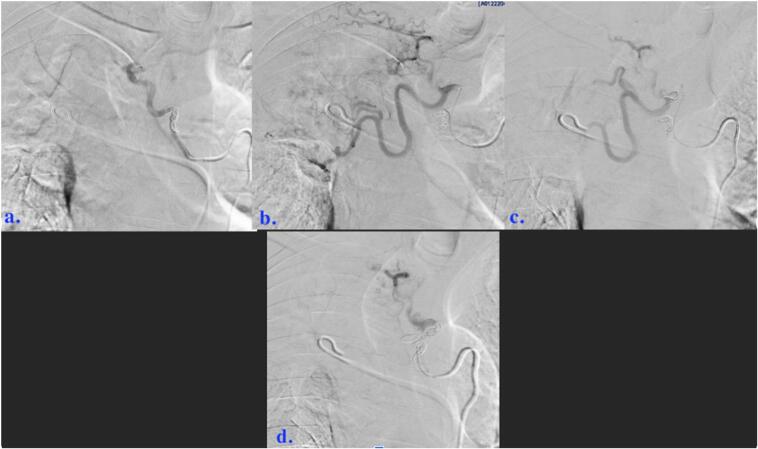
58-year-old male who experienced minimal hemoptysis for which investigations revealed the presence of a bronchopulmonary artery fistula successfully treated with coil embolization. (a through d) Purposeful embolizations of branches and communicators affecting BPAF.

A Progreat (Terumo Medical Corporation, Somerset, NJ, USA) microcatheter was advanced through and several centimeters beyond tip of the Mik catheter. 900–1200 μm Embosphere particles were delivered through this microcatheter (total four vials), followed by Ultrafoam (Becton, Dickenson and Company, Franklin Lanes, NJ, USA) slurry. 3 through 6 mm Tornado and/or Nestor (Cook Medical, Bloomington, IN, USA) microcoils were then deployed during sequential partial retractions of microcatheter. Satisfactory stasis was demonstrated following embolizations of two right bronchial arteries (congenital variant from more typical solitary right bronchial artery). High flow communicators remained patent and served as feeders of multi channeled fistula. These arose predominantly from right upper lobe bronchial artery branches. Each of these was selected with the microcatheter. Microcoil embolizations were performed prior to additional gelatin foam slurry boluses. Largest deployed microcoil expanded to 8 mm max (Fig. 5).

Two right bronchial arteries originated from descending thoracic aorta. Branches of both selected, stasis achieved. Follow up right pulmonary arteriography demonstrated reduced reversal of flow in the upper lobe branch.

The microcatheter was removed. Intercostal branches arising from descending thoracic aorta were subsequently selected with 5F catheter and examined by angiography but none of these warranted intervention. Hemoptysis ceased and the patient was discharged home following additional cardiac workup.

## 3 Discussion

Bronchopulmonary artery fistula (BPAF) most commonly occurs as a complication of lung transplantation or radiation therapy. BPAF may present as a congenital anomaly. Tuberculosis (TB) is the most common infectious etiology. Even among noninfectious contributors, coexisting TB involvement is often implicated. BPAF typically manifests years (perhaps decades) after documented initial TB onset [10].

Patients often present to emergency and urgent care facilities with signs or symptoms prompting concerns for pulmonary embolism. Chest CTA is typically obtained. Scanning is completed in a few seconds. Formal imaging is preceded by dedicated scout protocol. A contrast bolus is administered. Second-by-second imaging is obtained while keeping the CT table stationary at the level of bifurcation of the main pulmonary artery. The goal is to achieve maximal and uniform contrast enhancement of pulmonary arteries and their branches compared with remaining intrathoracic arterial and venous segments. Hypodense foci within otherwise uniformly opacified pulmonary arteries are usually correctly interpreted as pulmonary emboli. Scanning is performed often prior to appreciable or adequate opacifications of pulmonary veins, left heart, thoracic aorta, and great arteries. Bronchial arteries arise from the aorta, so these are also usually underopacified with CTA protocol to rule out PE. Intraluminal left ventricular and aortic forces normally far exceed those within the right ventricle and pulmonary arteries. Bronchopulmonary artery fistula (BPAF) flow is therefore from bronchial artery to pulmonary artery. If BPAF is situated in the right upper lobe, right upper lobe pulmonary artery branches will be at the outflow of any arterial fistula. Relatively unopacified blood from bronchial artery enters right pulmonary artery and causes ill-defined hypodensities mixing with opacified blood, especially compared to uniformly, brightly enhancing left pulmonary artery. Therefore interpreters will frequently incorrectly conclude that right pulmonary artery embolism exists.

BPAF is rare and therefore clear management guidelines are lacking. Embolization is commonly suggested as a treatment option. Bronchial artery embolization (BAE) halts massive hemoptysis in 81–100 % of cases [1]. Distal embolization with polyvinyl alcohol particles sized from 350 to 500 μm offers good results [11]. Gelfoam slurry, thrombin and glue are also acceptable options. Particles smaller than 200 μm and liquid embolics should be avoided to prevent tissue infarction [11]. Choosing appropriate treatment for hemoptysis caused by bronchopulmonary artery fistula (BPAF) can be challenging. Embolization is most commonly utilized but complications may be encountered. Inadvertent migration of embolized material and air embolus can be clinically significant [12]. Spinal artery embolization can of course be disastrous, resulting in paralysis. The choice of embolic agent therefore becomes crucial. Durability and size of the occlusion/occluded vessel and feasibility of delivery should be taken into consideration [4]. In cases of such shunts, placement of large particles and/or coils or may be used to prevent pulmonary or systemic emboli, as was done in our case [11,12].

Exclusive use of nonionic contrast, improved coaxial equipment such as microcatheters and microcoils, and better embolization particles have dramatically reduced complication rates. Risk is presently estimated at 0.1 % [1]. Other interventions are also available. Resecting concomitant aneurysms or lobectomy have effected cure [13]. Stent graft placement was utilized to manage BPAF in two cases to date [14,15]. Percutaneous direct puncture methods, as opposed to an endovascular approach, have been successful [16]. Of course a multidisciplinary team approach is essential in managing and/or treating this rare phenomenon.

The work has been reported in line with the SCARE criteria [17].

## 4 Conclusion

Patients presenting with hemoptysis, whether massive or nonmassive, will often undergo chest CTA. Hemodynamics in cases of bronchopulmonary artery fistula will result in multiple areas of unopacified blood in communicating pulmonary arterial segments. Misdiagnosis of pulmonary embolism will therefore commonly ensue. These arterial fistulas are usually treated by bronchial artery embolization. Direct puncture or stent grafting are alternate considerations depending on anatomic presentation. Open surgery may be necessary especially if percutaneous methods have already been utilized. In all instances, a multidisciplinary approach is a must.

Learning points:

• Bronchopulmonary artery fistula (BPAF), reported in 2.6–13 % of cases of hemoptysis, most commonly occurs as a complication of lung transplantation or radiation therapy and the most common infectious cause includes tuberculosis.
• Bronchial arteries arise from the aorta, so these are also usually underopacified with CTA protocol to rule out PE.
• Embolization is most commonly utilized to treat BPAF; however, spinal artery embolization may occur, resulting in paralysis.
• Other interventions include lobectomy, stent graft placement and percutaneous direct puncture in the treatment of BPAF.

## Consent

Informed consent was obtained from the patient before writing up this case study.

## Ethical approval

Waived as per hospital policy.

## Funding

None to report.

## Guarantor

Shivam Khatri.

## CRediT authorship contribution statement

Shivam Khatri: study concept, data collection, data analysis, writing of the paper
Steven Epstein: study concept, data collection, data analysis, writing of the paper
Rooshi Parikh: writing of the paper
Bobby Chiong: writing of the paper.

## Declaration of competing interest

None to report.
